# Phase 2 randomized controlled trial of intravenous or intraperitoneal paclitaxel plus mFOLFOX6 vs. mFOLFOX6 as first-line treatment of advanced gastric cancer

**DOI:** 10.3389/fonc.2022.850242

**Published:** 2022-09-07

**Authors:** Shen Zhao, Liyu Su, Yigui Chen, Xiaofeng Li, Peicheng Lin, Wujin Chen, Wenzheng Fang, Jinfeng Zhu, Hui Li, Liping Ren, Jie Liu, Yanni Hong, Shaowei Lin, Nanfeng Fan, Rongbo Lin

**Affiliations:** ^1^ Department of Gastrointestinal Medical Oncology, Clinical Oncology School of Fujian Medical University, Fujian Cancer Hospital, Fuzhou, China; ^2^ Fujian Key Laboratory of Translational Cancer Medicine, Fuzhou, China; ^3^ Department of Medical Oncology, Quanzhou First Hospital, Quanzhou, China; ^4^ Department of Radiation Oncology, Clinical Oncology School of Fujian Medical University, Fujian Cancer Hospital, Fuzhou, China; ^5^ Department of Medical Oncology, Affiliated People’s Hospital of Fujian University of Traditional Chinese Medicine, Fuzhou, China; ^6^ Department of Medical Oncology, The 900th Hospital of Joint Logistic Support Force People’s Liberation Army, Fuzhou, China; ^7^ School of Public Health, Fujian Medical University, Fuzhou, China

**Keywords:** advanced, gastric cancer, intraperitoneal, intravenous, paclitaxel

## Abstract

**Objective:**

We conducted a phase 2 trial to compare the safety and efficacy of intravenous paclitaxel or intraperitoneal paclitaxel plus mFOLFOX6 vs. mFOLFOX6 in untreated advanced gastric cancer.

**Methods:**

Participants with untreated advanced gastric cancer were randomly assigned (1:1:1) to: intravenous paclitaxel 135 mg/m^2^ or intraperitoneal paclitaxel 80 mg/m^2^ plus mFOLFOX6 omitting bolus fluorouracil; or mFOLFOX6 (oxaliplatin 85 mg/m^2^, leucovorin 400 mg/m^2^, fluorouracil 400 mg/m^2^ bolus, fluorouracil 2,400 mg/m^2^ 46-h continuous infusion). Treatment was every 14 days for up to 9 cycles followed by S-1 maintenance. The primary outcome was progression-free survival.

**Results:**

Of 90 enrolled participants, 30 in the intravenous paclitaxel group, 29 in the intraperitoneal paclitaxel group, and 30 in the mFOLFOX6 group were included in the analyses. The median progression-free survival was 6.52, 5.83, and 4.55 months, respectively, for the intravenous paclitaxel group, intraperitoneal paclitaxel group, and mFOLFOX6 group. The hazard ratios were 0.56 (95% CI: 0.33–0.94; *p* = 0.026) and 0.56 (95% CI: 0.33–0.96; *p* = 0.037), respectively, for the intravenous paclitaxel group and the intraperitoneal paclitaxel group vs. the mFOLFOX6 group. The most common grade 3/4 adverse events for the intravenous paclitaxel group, intraperitoneal paclitaxel group, and mFOLFOX6 group, respectively, were neutropenia (30.0%, 34.5%, 33.3%), diarrhea (13.3%, 20.7%, 13.3%), and leukopenia (10.0%, 13.8%, 10.0%). No treatment-related death occurred.

**Conclusion:**

The findings of this phase 2 trial suggest that adding intravenous paclitaxel or intraperitoneal paclitaxel to mFOLFOX6 for untreated advanced gastric cancer improved progression-free survival with manageable adverse events.

## Introduction

China has one of the world’s highest incidence rates of gastric cancer (GC), accounting for 42.6% of global incidence (1–3). Although GC in China has decreased in recent years, it remains the second most common cancer among men and the third most common among women (2, 3). While surgery with or without pre- or postoperative chemotherapy is the only potential curative treatment for early-stage GC (3), more than 80% of patients present with advanced GC (AGC), particularly in rural areas (2, 3).

The safety and efficacy of doublet regimens including fluoropyrimidine combined with either oxaliplatin or cisplatin have been widely reported (4, 5) and are recommended for untreated AGC (uAGC) (3, 6, 7). However, for fit patients, triplet chemotherapy also has been recommended (3, 6, 7). The V325 phase 3 trial reported that docetaxel, cisplatin, and fluorouracil (DCF) significantly improved time to progression, overall survival (OS), and overall response rate (ORR) compared with cisplatin and fluorouracil (CF) in uAGC. However, DCF has not been accepted globally due to its severe myelosuppression and small survival advantage (8). Various modifications of the DCF regimen, including intravenous (IV) paclitaxel, oxaliplatin, fluorouracil, and leucovorin (ivPOF) demonstrated improved safety, which was validated in Chinese patients with AGC (9–11). However, a randomized phase 3 trial showed no significant difference in progression-free survival (PFS), OS, or ORR between doublet irinotecan, fluorouracil, and leucovorin (FOLFIRI) and triplet epirubicin, cisplatin, and fluorouracil (ECF), with less toxicity and better tolerance attributed to FOLFIRI (12). Nonetheless, some studies suggest that DCF is superior to ECF (13, 14,), and controversy remains concerning triplet vs. doublet therapy in uAGC.

Peritoneal involvement, the most frequent site of metastasis in AGC, confers a dismal prognosis (3, 6, 7). Compared with plasma clearance, peritoneal clearance of certain hydrophobic and high-molecular-weight agents, such as paclitaxel, is much slower (15, 16). Therefore, intraperitoneal (IP) paclitaxel is designed to target peritoneal tumor nodules while minimizing systemic toxicity (15–20). The phase 2 studies of IP paclitaxel with S-1 and IV paclitaxel showed promising results, with a median OS of 17.6–23.6 months and ORR of 56%–71% (17–19). However, dosages of IP paclitaxel (recommended: 20 mg/m^2^ to 80 mg/m^2^) are controversial (15, 16). We conducted a phase 1b study comparing IP paclitaxel 60 mg/m^2^ day 1 plus modified ivPOF (IV paclitaxel 100 mg/m^2^) with IP paclitaxel 80 mg/m^2^ day 1 plus mFOLFOX6 (oxaliplatin, fluorouracil, and leucovorin). Both dose levels of IP paclitaxel were well-tolerated (published at the 2017 meeting of the Chinese Society of Clinical Oncology). IP paclitaxel 80 mg/m^2^ day 1 plus mFOLFOX6 (ipPOF) was selected for the current trial with ivPOF to clearly delineate their safety and efficacy compared with mFOLFOX6 in participants with uAGC.

## Materials and methods

### Study design

**Figure 1:**
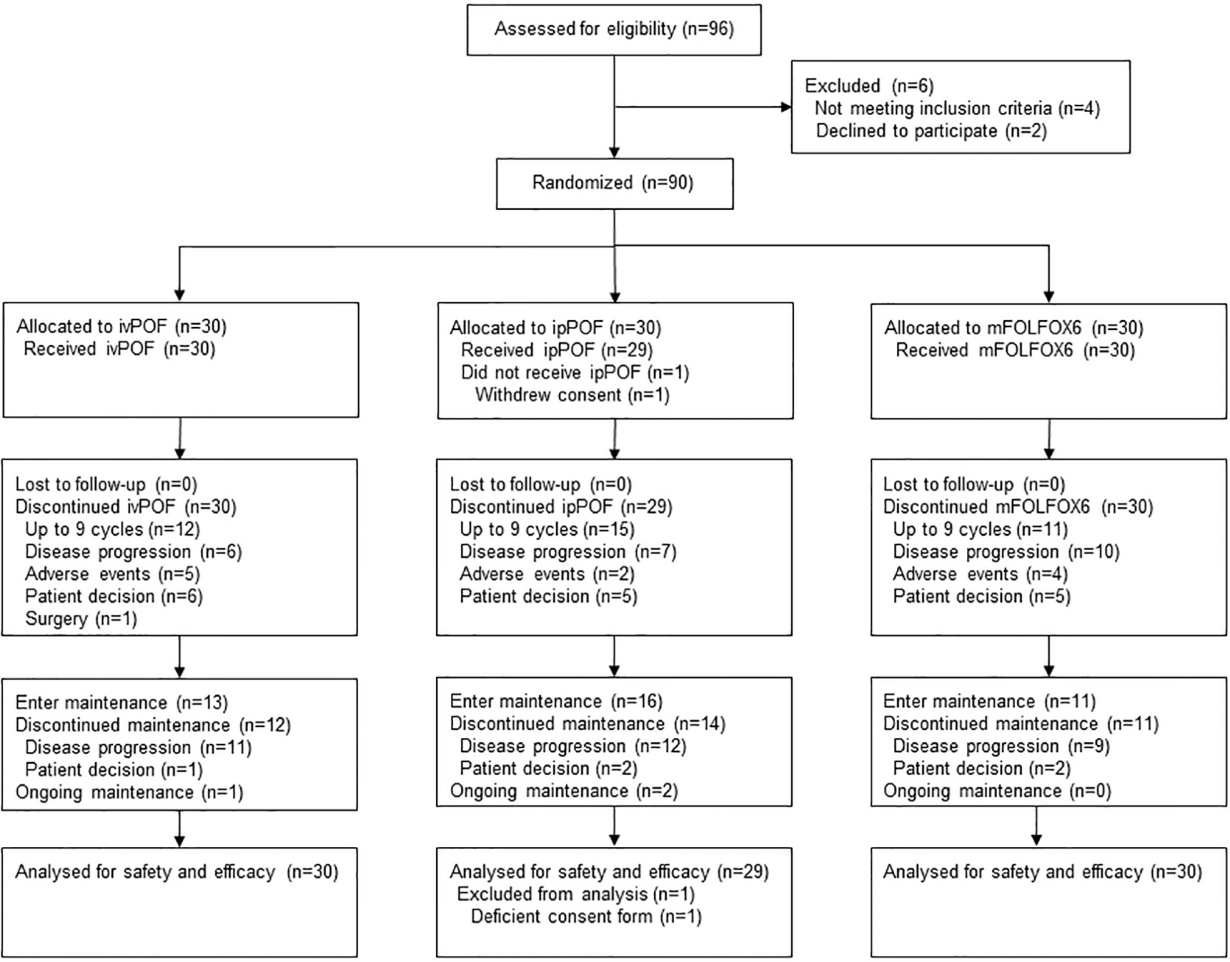
Trial profile. ivPOF, intravenous paclitaxel, oxaliplatin, fluorouracil, and leucovorin; ipPOF, intraperitoneal paclitaxel, oxaliplatin, fluorouracil, and leucovorin; mFOLFOX6, modified oxaliplatin, fluorouracil, and leucovorin.

SYLT/FNF-004 is a multicenter, randomized, parallel, open-label, phase 2 trial conducted at six oncology centers in China. Subjects were randomly assigned (1:1:1) to receive ivPOF, ipPOF, or mFOLFOX6 (**Figure 1**) after providing written informed consent. There were no stratification criteria for randomization. The protocol was approved by the central ethics committee of the Fujian Cancer Hospital and the local ethics committees of all participating hospitals and was conducted in accordance with the principles of the Declaration of Helsinki and Good Clinical Practice. All participants provided written informed consent.

### Participants

Eligibility criteria included: histologically or cytologically confirmed metastatic or unresectable gastric or gastroesophageal junction adenocarcinoma with measurable disease according to the Response Evaluation Criteria in Solid Tumors (RECIST) guidelines (version 1.1) (21), no prior therapy except neo- or adjuvant chemotherapy completed ≥6 months prior to relapse, age 18 to 75 years, Eastern Cooperative Oncology Group performance status (ECOG PS) 0 or 1, and adequate organ function. Exclusion criteria included: ascites requiring frequent drainage, peripheral neuropathy grade ≥2 according to the National Cancer Institute Common Terminology Criteria for Adverse Events (NCI-CTCAE) version 4.03, brain or leptomeningeal involvement, concurrent cancer, or uncontrolled significant comorbidities. When the SYLT/FNF-004 trial was designed, trastuzumab was not yet covered by medical insurance and was not affordable to most Chinese patients; those who did not intend to use trastuzumab were allowed study entry regardless of human epidermal growth factor receptor 2 (HER2) status. Peritoneal metastasis (PM) diagnosed by ultrasound, computed tomography, magnetic resonance imaging, positron emission tomography, or ascites was designated as macroscopic PM (MAPM). MAPM was not a mandatory eligibility criterion.

### Randomization and masking

Before the start of the study, a computer-generated sequence of random numbers was placed in a series of plain, sealed envelopes with patient numbers on them for the research nurse. These envelopes were created and kept at the School of Public Health of Fujian Medical University and only opened at the time of subject randomization, again by the research nurse. Individuals directly involved in the study had no access to these envelopes. Subjects were randomized into blocks of three. Subjects had an equal chance of being assigned to the groups. The statistician and research nurse are blinded to the recruitment procedure prior to the initiation of the trial. Because this was an open-label trial, the patients and physicians were not masked from the study groups. A site radiologist who assessed tumor radiographic responses, a central radiologist who verified them, and a statistician who statistically analyzed the data were blinded to the study groups.

### Procedures

Participants in the mFOLFOX6 group received induction treatment consisting of a 2-h infusion of oxaliplatin at 85 mg/m^2^ plus leucovorin at 400 mg/m^2^, followed by a fluorouracil bolus of 400 mg/m^2^ and a 46-h infusion of fluorouracil at 2,400 mg/m^2^. Those assigned to ivPOF or ipPOF received a 3-h infusion of IV paclitaxel at 135 mg/m^2^ or IP paclitaxel at 80 mg/m^2^ followed by mFOLFOX6 (omitting the fluorouracil bolus). A central venous catheter was indwelled into the abdominal cavity before the administration of IP paclitaxel, which was diluted in normal saline to 500 ml. Normal saline perfusion of 500 ml was planned to be given before and after paclitaxel (total 1,000 ml) but was reduced for ascites, accordingly. The catheter was removed 2 days after treatment administration. Induction treatment was repeated every 14 days for up to 9 cycles. Thereafter, the investigator determined whether to use maintenance therapy (S-1 80 mg/m^2^ days 1–14, repeated every 3 weeks). Induction or maintenance therapy continued until progressive disease (PD), unacceptable toxicity, subject refusal, or investigator decision. Antiemetic prophylaxis was given according to local protocols; granulocyte colony-stimulating factor was not recommended as primary prophylaxis. Premedications (antihistamine, corticosteroid, and H2 receptor antagonist) were administered for prophylaxis of hypersensitivity reactions to IV or IP paclitaxel. Doses were modified in response to toxicities (**Supplementary Material S1**). Patients with dose interruptions for more than 4 weeks should permanently discontinue the treatment. Laboratory studies to monitor bone marrow, liver, and kidney function were done within 7 days of randomization and up to 2 days before each treatment after the first treatment cycle. Tumor assessment by computed tomography or magnetic resonance imaging was performed every 6 weeks until evidence of PD was detected. After PD, participants were contacted every 12 weeks to assess survival and obtain information on subsequent treatment. Adverse events (AEs), including serious adverse events, were monitored throughout the study period until resolved, returned to baseline, or deemed irreversible, or until lost to follow-up or study withdrawal by participant or investigator. HER2 status was assessed by immunohistochemistry or fluorescence *in situ* hybridization using biopsy or surgical specimens. HER2 positivity is defined as immunohistochemistry 3+ or as immunohistochemistry 2+ plus fluorescence *in situ* hybridization positive (HER2:CEP17 ratio ≥2). HER2 negativity is defined as immunohistochemistry 0 or 1+ or as immunohistochemistry 2+ plus fluorescence *in situ* hybridization negative (22, 23).

### Outcomes

The primary outcome was PFS, defined as the time from treatment assignment to documented PD per RECIST version 1.1 according to investigator assessment, or death from any cause, whichever occurred first. Secondary outcomes included OS (time from treatment assignment to death from any cause), best overall tumor response from baseline per RECIST version 1.1, and AEs graded according to the NCI-CTCAE version 4.03.

### Statistical analysis

On the basis of previous studies and our clinical practice (4, 5, 11, 17–19), we expected a median PFS of 7 months in either the ivPOF or ipPOF arm and 4 months for mFOLFOX6. The chosen sample size was calculated by a log-rank test based on the primary endpoint to verify the superiority of ivPOF or ipPOF vs. mFOLFOX6. A two-sided *α* of 0.05 was used, with 0.025 allocated to the hypothesis of ivPOF vs. mFOLFOX6 or ipPOF vs. mFOLFOX6, separately. We calculated that 54 subjects in each arm were needed, over 24 months of accrual and 24 months of follow-up, to achieve 80% statistical power for each hypothesis. Considering a dropout rate of 10%, the total number to be enrolled was 178. The full dataset for efficacy and safety analyses included all randomly assigned participants who received at least one dose of study medication. Categorical variables are presented as frequencies/proportions, and continuous variables as medians/interquartile ranges (IQRs). Between-group differences were analyzed with the *χ* ^2^ test and Fishers’ exact test. We estimated PFS and OS using Kaplan–Meier with a *p*-value. Hazard ratios (HRs) and 95% confidence intervals (CIs) were estimated using Cox proportional hazards model. *p* < 0.05 is considered statistically significant. The data were analyzed using R software, version 4.0.

## Results

A total of 96 patients were screened and 90 (93.8%) were randomly assigned to receive ivPOF (*n* = 30), ipPOF (*n* = 30), or mFOLFOX6 (*n* = 30) at six oncology centers in China between 30 November 2015 and 21 May 2018 (**Figure 1**). The trial was closed due to poor accrual. One participant in the ipPOF group did not receive any study medication after randomization and was excluded from efficacy and safety analyses. The patient’s baseline characteristics were well-balanced (**Table 1**). Most patients (88.9%, 80/90) were HER2 negative. The median number of cycles administered/participant was 6 (IQR, 4 to 9) for ivPOF, 9 (IQR, 4 to 9) for ipPOF, and 4 (IQR, 3 to 9) for mFOLFOX6. Two participants in the ivPOF group and one in the mFOLFOX6 group received >9 cycles, considered protocol violations. Treatment was more often delayed in the ivPOF group (15.1%) compared with the ipPOF (8.2%; *p* = 0.034) or mFOLFOX6 (5.5%; *p* = 0.004). Doses were more frequently reduced in the ivPOF group (18.8%) compared with the ipPOF (9.2%; *p* = 0.007) or mFOLFOX6 (7.3%; *p* = 0.002). In the ivPOF group, average dose intensities of paclitaxel, oxaliplatin, and fluorouracil were 86.6%, 86.7%, and 87.4%, respectively, compared with 92.8%, 91.9%, and 92.3%, respectively, for ipPOF. In the mFOLFOX6 group, the average dose intensities of oxaliplatin and fluorouracil were 93.5% and 93.9%, respectively.

**Table 1:** Patient demographics and baseline characteristics.

Characteristic	ivPOF	ipPOF	mFOLFOX6
Participants (*n*)	30	29	30
Age (years)			
Median (IQR)	58.5 (40, 64)	52 (44, 62)	59.5 (46, 69)
Male [*n* (%)]	22 (73.3)	14 (48.3)	18 (60.0)
ECOG PS [*n* (%)]			
0	14 (46.7)	13 (44.8)	10 (33.3)
1	16 (53.3)	16 (55.2)	20 (66.7)
Histologic type^a^ [*n* (%)]			
Differentiated	7 (23.3)	4 (13.8)	6 (20.0)
Undifferentiated	19 (63.3)	23 (79.3)	18 (60.0)
Unknown	4 (13.3)	2 (6.9)	6 (20.0)
Disease status [*n* (%)]			
Initially metastatic	21 (70.0)	14 (48.3)	18 (60.0)
Postgastrectomy	9 (30.0)	15 (51.7)	12 (40.0)
Metastatic sites [*n* (%)]			
1	14 (46.7)	15 (51.7)	12 (40.0)
≥2	16 (53.3)	14 (48.3)	18 (60.0)
Organs involved [*n* (%)]			
Primary tumor site	21 (70.0)	15 (51.7)	16 (53.3)
Peritoneum	12 (40.0)	17 (58.6)	16 (53.3)
Abdominal cavity lymph nodes	20 (66.7)	12 (41.4)	22 (73.3)
Lymph nodes	21 (70.0)	13 (44.8)	23 (76.7)
Liver	9 (30.0)	8 (27.6)	7 (23.3)
Lung	4 (13.3)	2 (6.9)	0
Ovary	1 (3.3)	3 (10.3)	5 (16.7)
Bone	4 (13.3)	1 (3.4)	3 (10.0)
Soft tissue	1 (3.3)	0	0
Adrenal gland	0	0	1 (3.3)
Prior chemotherapy^b^ [*n* (%)]	5 (16.7)	3 (10.3)	6 (20.0)
HER2 positive^c^ [*n* (%)]	3 (10.0)	4 (13.8)	3 (10.0)

a Differentiated = 1, papillary adenocarcinoma and tubular adenocarcinoma (well differentiated, moderately differentiated); undifferentiated = 2, poorly differentiated adenocarcinoma (solid type, nonsolid type), signet ring cell carcinoma, and mucinous adenocarcinoma.

b Prior chemotherapy refers to recurrence or metastasis >6 months following neoadjuvant or adjuvant chemotherapy.

c HER2 positivity is defined as immunohistochemistry 3+ or as immunohistochemistry 2+ plus fluorescence in-situ hybridization positive (HER2:CEP17 ratio≥2).

ECOG PS, Eastern Cooperative Oncology Group Performance Status; ivPOF, intravenous paclitaxel, oxaliplatin, fluorouracil, and leucovorin; ipPOF, intraperitoneal paclitaxel, oxaliplatin, fluorouracil, and leucovorin; mFOLFOX6, modified oxaliplatin, fluorouracil, and leucovorin; IQRs, interquartile ranges; HER2, human epidermal growth factor receptor 2.

The main reason for induction discontinuation for ivPOF, ipPOF, and mFOLFOX6 was the completion of 9 cycles: ivPOF, 12/30 (40.0%); ipPOF, 15/29 (51.7%); and mFOLFOX6, 11/30 (36.7%). Thirteen (43.3%) patients in the ivPOF group, 16 (51.2%) in the ipPOF group, and 11 (36.7%) in the mFOLFOX6 group received S-1 as maintenance (**Figure 1**). One patient in the ivPOF group began maintenance prior to completing 9 cycles due to an AE. The main reason for maintenance discontinuation for ivPOF, ipPOF, and mFOLFOX6, respectively, was PD: 11 patients (84.6%), 12 patients (75.0%), and 9 patients (81.8%).

**Figure 2:**
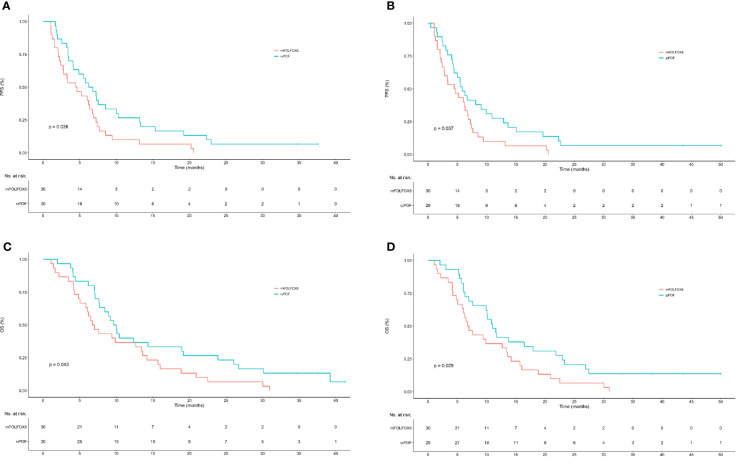
Kaplan–Meier estimates of PFS and OS for ivPOF or ipPOF vs. mFOLFOX6. **(A)** Median PFS (months) was 6.52 (95% CI: 4.13–10.27) in the ivPOF group and 4.55 (95% CI: 2.73–6.87) in the mFOLFOX6 group (HR, 0.56; 95% CI: 0.33–0.94; *p* = 0.026). **(B)** Median PFS (months) was 5.83 (95% CI: 4.43–10.93) in the ipPOF group and 4.55 (95% CI: 2.73–6.87) in the mFOLFOX6 group (HR, 0.56; 95% CI: 0.33–0.96; *p* = 0.037). **(C)** Median OS (months) was 9.83 (95% CI: 7.70–19.2) in the ivPOF group and 6.87 (95% CI: 5.83–13.6) in the mFOLFOX6 group (HR, 0.59; 95% CI: 0.35–1.00; *p* = 0.043). **(D)** Median OS (months) was 11.03 (95% CI: 9.93–21.8) in the ipPOF group and 6.87 (95% CI: 5.83–13.6) in the mFOLFOX6 group (HR, 0.54; 95% CI: 0.32–0.029). PFS, progression-free survival; OS, overall survival; ivPOF, intravenous paclitaxel, oxaliplatin, fluorouracil, and leucovorin; mFOLFOX6, modified oxaliplatin, fluorouracil, and leucovorin; CI, confidence interval; HR, hazard ratio.

As of 31 December 2020, data cutoff, median follow-up (months) was 41 (IQR, 37 to 43). Compared with ivPOF or ipPOF separately, mFOLFOX6 demonstrated positive results for PFS and OS. Median PFS (months) and OS (months) for ivPOF were 6.52 (95% CI: 4.13–10.27) and 9.83 (95% CI: 7.70–19.2); for ipPOF, they were 5.83 (95% CI: 4.43–10.93) and 11.03 (95% CI: 9.93–21.8); and for mFOLFOX6, they were 4.55 (95% CI: 2.73–6.87) and 6.87 (95% CI: 5.83–13.6). For PFS, ivPOF vs. mFOLFOX6 HR = 0.56 (95% CI: 0.33–0.94; *p* = 0.026; **Figure 2A**); ipPOF vs. mFOLFOX6 HR = 0.56 (95% CI: 0.33–0.96; P=0.037; **Figure 2B**). For OS, ivPOF vs. mFOLFOX6 HR = 0.59 (95% CI: 0.35–1.00; *p* = 0.043; **Figure 2C**); ipPOF vs. mFOLFOX6 HR = 0.54 (95% CI: 0.32–0.93; *p* = 0.029; **Figure 2D**). Response rates were 17/30 (56.7%; 95% CI: 38.9–74.4) for ivPOF and 11/29 (37.9%, 95% CI: 20.3–55.6) for ipPOF. Compared with mFOLFOX6, these were not significantly different (**Table 2**).

**Table 2:** Best overall response rate.

Response (*n* (%)	ivPOF (*n* = 30)	ipPOF (*n* = 29)	mFOLFOX6 (*n* = 30)
Complete response	4 (13.3%)	2 (6.9%)	2 (6.7%)
Partial response	13 (43.3%)	9 (31.0%)	9 (30.0%)
Response rate	17 (56.7%)	11 (37.9%)	11 (36.6%)
95% CI	38.9–74.4	20.3–55.6	38.9–74.4
Stable disease	9 (30.0%)	12 (41.4%)	12 (40%)
Progressive disease	3 (10.0%)	6 (20.7%)	6 (20.0%)
Not evaluable	1 (3.3%)	0	1 (3.3%)

p = 0.121 for ivPOF vs. mFOLFOX6; p = 0.920 for ipPOF vs. mFOLFOX6; p = 0.150 for ivPOF vs. ipPOF.

ivPOF, intravenous paclitaxel, oxaliplatin, fluorouracil, and leucovorin; ipPOF, intraperitoneal paclitaxel, oxaliplatin, fluorouracil, and leucovorin; mFOLFOX6, modified oxaliplatin, fluorouracil, and leucovorin; CI, confidence interval.

**Figure 3:**
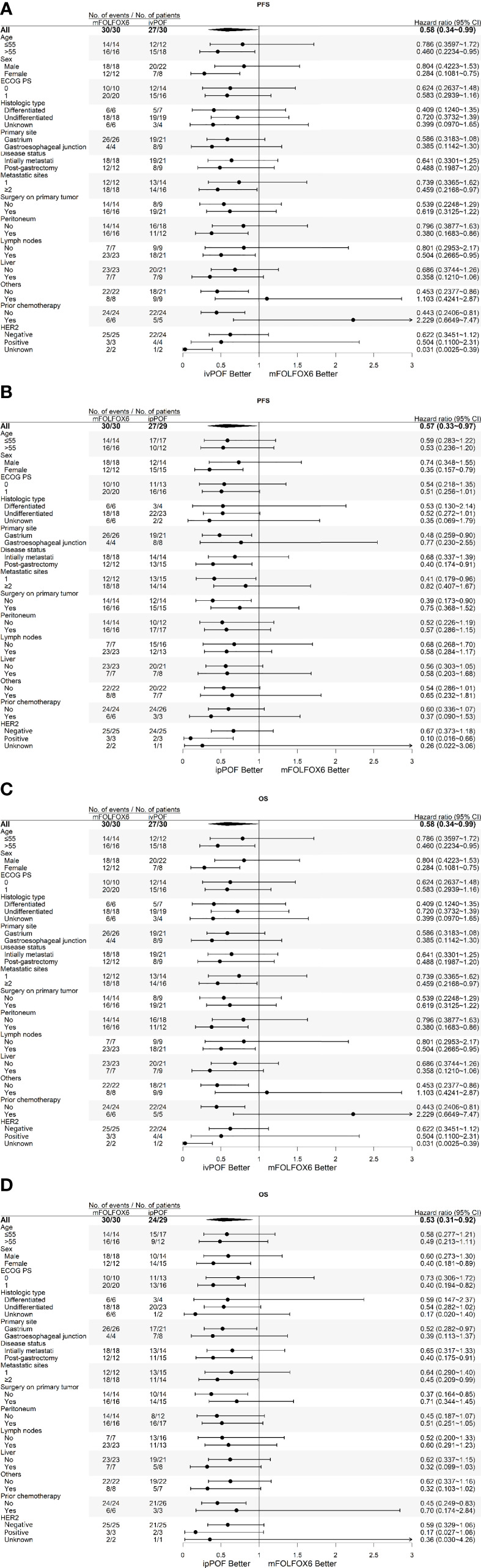
Subgroup analyses of PFS for **(A)** ivPOF vs. mFOLFOX6 and **(B)** ipPOF vs. mFOLFOX6 or OS for **(C)** ivPOF vs. mFOLFOX6 and **(D)** ipPOF vs. mFOLFOX6. HER2 positivity is defined as immunohistochemistry 3+ or as immunohistochemistry 2+ plus fluorescence *in situ* hybridization positive (HER2:CEP17 ratio ≥2), HER2 negativity is defined as immunohistochemistry 0 or 1+ or as immunohistochemistry 2+ plus fluorescence *in situ* hybridization negative. PFS, progression-free survival; OS, overall survival; ivPOF, intravenous paclitaxel, oxaliplatin, fluorouracil, and leucovorin; ipPOF, intraperitoneal paclitaxel, oxaliplatin, fluorouracil, and leucovorin; mFOLFOX6, modified oxaliplatin, fluorouracil, and leucovorin; HR, hazard ratio.

The exploratory *post-hoc* subgroup analyses of PFS or OS according to baseline demographics and disease characteristics consistently favored ivPOF or ipPOF over mFOLFOX6 (**Figure 3**). Median PFS and OS is 4.83 months (95% CI: 3.19, 7.30) vs. 6.13 months (95% CI: 4.93, 7.56, *p* = 0.121) and 8.84 months (95% CI: 6.64, 11.41) vs. 11.54 months (95% CI: 8.32, 18.7, *p* = 0.251) in MAPM vs. non-MAPM, respectively. The therapeutic efficacy for HER2-positive subjects is displayed in **Table 3**. Patients 6 and 7 went on to a phase 2 trial of RC48-ADC (HER2-targeting antibody-drug conjugate).

**Table 3:** Therapeutic efficacy in HER2-positive subjects.

Subject	Group	Best response	PFS (months)	OS (months)
1	ivPOF	SD	7.20	9.53
2	ivPOF	SD	2.53	6.90
3	ivPOF	PR	9.86	9.86
4	ipPOF	CR	43.00^a^	43.00^a^
5	ipPOF	SD	8.94	11.54
6	ipPOF	PR	8.02	37.71^a^
7	ipPOF	SD	12.72	32.81^a^
8	mFOLFOX6	SD	2.37	4.18
9	mFOLFOX6	PD	1.55	6.41
10	mFOLFOX6	SD	4.41	6.90

a No event occurred until the cutoff date.

PFS, progression-free survival; OS, overall survival; ivPOF, intravenous paclitaxel, oxaliplatin, fluorouracil, and leucovorin; ipPOF, intraperitoneal paclitaxel, oxaliplatin, fluorouracil, and leucovorin; mFOLFOX6, modified oxaliplatin, fluorouracil, and leucovorin; CR, complete response; PR, partial response; SD, stable disease; PD, progressive disease.

Reports of treatment-emergent AEs (any grade) were similar (ivPOF, 93.3%; ipPOF, 100%; and mFOLFOX6, 93.3%). The frequency of grade 3 or 4 AEs were also similar (ivPOF, 50.0%; ipPOF, 51.7%; and mFOLFOX6, 56.7%). The most common grade 3 or 4 AEs were neutropenia, diarrhea, leukopenia, fatigue, and sensory neuropathy (**Table 4**). There was no between-group difference in all-grade or grade 3 or 4 toxicity, except for increased all-grade alanine aminotransferase in ivPOF. One subject had subcutaneous tumor implantation with a poor response to chemotherapy in the ipPOF group. All five allergic reactions were attributed to oxaliplatin (ivPOF, 3; ipPOF, 1; mFOLFOX6, 1). Grade 1 or 2 abdominal pain occurred in six patients in the ipPOF group, induced by catheter stimulation, which was resolved by repositioning the catheter. No unexpected serious adverse event or protocol-related death occurred.

**Table 4:** Adverse events.

	ivPOF (*n* = 30)		ipPOF (*n* = 29)		mFOLFOX6 (*n* = 30)	
Toxicity [*n* (%)]	All grades	Grade 3 or 4	All grades	Grade 3 or 4	All grades	Grade 3 or 4
Any event	28 (93.3)	15 (50.0)	29 (100)	15 (51.7)	28 (93.3)	17 (56.7)
Leukopenia	20 (66.7)	3 (10.0)	19 (65.5)	4 (13.8)	18 (60.0)	3 (10.0)
Neutropenia	18 (60.0)	9 (30.0)	17 (58.6)	10 (34.5)	17 (56.7)	10 (33.3)
Febrile neutropenia	1 (3.3)	1 (3.3)	1 (3.4)	1 (3.4)	0	0
Anemia	18 (60.0)	1 (3.3)	19 (65.5)	1 (3.4)	15 (50.0)	0
Thrombocytopenia	7 (23.3)	0	7 (24.1)	0	4 (13.3)	1 (3.3)
Hyperbilirubinemia	2 (6.7)	0	5 (17.2)	0	4 (13.3)	0
Alanine aminotransferase increased	13 (43.3)	0	4 (13.8)	0	6 (20.0)	0
Creatinine increased	0	0	1 (3.4)	0	1 (3.3)	0
Fatigue	24 (80.0)	3 (10.0)	22 (75.9)	2 (6.9)	24 (80.0)	1 (3.3)
Anorexia	19 (63.3)	2 (6.7)	22 (75.9)	1 (3.4)	21 (70.0)	1 (3.3)
Nausea	12 (40.0)	2 (6.7)	16(55.2)	0	19 (63.3)	0
Vomiting	6 (20.0)	1 (3.3)	4 (13.8)	0	7 (23.3)	0
Diarrhea	11 (36.7)	4 (13.3)	11 (37.9)	6 (20.7)	9 (30.0)	4 (13.3)
Sensory neuropathy	18 (60.0)	3 (10.0)	13 (44.8)	1 (3.4)	15 (50.0)	3(3.3)
Stomatitis	6 (20.0)	1 (3.3)	7 (24.1)	0	6 (20.0)	1 (3.3)
Hand–foot syndrome	3 (10.0)	0	2 (6.9)	0	4 (13.3)	0
Myalgia	4 (13.3)	0	4 (13.9)	1 (3.4)	2 (6.7)	0
Allergic reaction	3 (10.0)	0	1 (3.4)	0	1 (3.3)	0
Abdominal pain induced by catheter	0	0	6 (20.7)	0	0	0

ivPOF, intravenous paclitaxel, oxaliplatin, fluorouracil, and leucovorin; ipPOF, intraperitoneal paclitaxel, oxaliplatin, fluorouracil, and leucovorin; mFOLFOX6, modified oxaliplatin, fluorouracil, and leucovorin.

Twelve (40.0%), 19 (65.5%), and 19 (63.3%) patients in the ivPOF, ipPOF, and mFOLFOX6 arms, respectively, received tumor-related drug therapy after completing the study treatment. Details of late-line treatments are displayed in **Table 5**. Five participants underwent curative intervention (**Table 6**).

**Table 5:** Subsequent chemotherapy.

Treatment regimen	ivPOF (*n* = 30)	ipPOF (*n* = 29)	mFOLFOX6 (*n* = 30)
Second-line (*n*)	12	19	19
APA+PAC+OXA+FU/LV	2	0	0
PAC/DOC+OXA+FU/LV	4	2	1
DOC+S-1+APA	0	0	1
FOLFOX+ipPAC	0	3	0
FOLFOX+APA	0	0	1
FOLFOX	0	0	1
FOLFIRI	2	5	1
DOC/PAC+S-1/FU/LV	0	8	11
APA+S-1	1	0	0
APA+DOC	0	0	2
APA	1	0	0
S-1	1	0	1
IRI	1	0	0
RC48-ADC	0	1	0
Third-line (*n*)	5	12	8
APA+PAC+OXA+FU/LV	1	1	1
APA+FOLFIRI+ipPAC	0	0	1
PAC+OXA+FU/LV	1	2	0
FOLFIRI+ipPAC	1	0	0
PAC+CAP+APA	0	1	0
FOLFIRI	0	1	1
DOC+RAL	0	1	0
APA+S-1	1	0	0
ANA+SIN	1	0	0
IRI+RAL	0	2	0
DOC+S-1	0	2	2
S-1	0	0	1
CAP	0	0	1
APA	0	1	1
RC48-ADC	0	1	0
Fourth-line (*n*)	3	3	2
APA+PAC+OXA+FU/LV	0	0	1
PAC+RAL+APA+SIN	0	1	0
FOLFIRI+SIN	1	0	0
Nab-PAC+OXA+SIN	1	0	0
FOLFIRI	0	1	1
DOC+SIN	0	1	0
Nab-PAC+OXA	1	0	0
Fifth-line (*n*)	0	1	0
DOC+RAL+APA+SIN	0	1	0

APA, apatinib; PAC, paclitaxel; OXA, oxaliplatin; FU/LV, fluorouracil/leucovorin; FOLFIRI, fluorouracil/leucovorin/irinotecan; DOC, docetaxel; IRI, irinotecan; SIN, sintilimab; ipPAC, intraperitoneal paclitaxel; ANA, anlotinib; FOLFOX, fluorouracil/leucovorin/oxaliplatin; RAL, raltitrexed; CAP, capecitabine; RC48-ADC, a novel, investigational, HER2-targeting antibody-drug conjugate.

**Table 6:** Therapeutic efficacy in subjects who underwent curative intervention.

Subject	Group	Curative intervention	Best response before intervention	PFS (months)	OS (months)
1	ivPOF	SBRT for liver metastases	PR	15.12	40.70^a^
2	ivPOF	RFA for liver metastases	PR	6.15	29.72
3	ivPOF	Total gastrectomy with D2 lymphadenectomy	PR	34.26^a^	34.26^a^
4	ivPOF	EBRT for retroperitoneal lymph nodes	PR	18.97	25.71
5	mFOLFOX6	Total gastrectomy with D2 lymphadenectomy	PR	6.12	12.49

a No event occurred until the cutoff date.

SBRT, stereotactic body radiotherapy; RFA: radiofrequency ablation; EBRT, external beam radiotherapy; PFS, progression-free survival; OS, overall survival; PR, partial response; ivPOF, intravenous paclitaxel, oxaliplatin, fluorouracil, and leucovorin; mFOLFOX6, modified oxaliplatin, fluorouracil, and leucovorin.

## Discussion

Our study shows that, compared with mFOLFOX6, IV or IP paclitaxel plus mFOLFOX6 significantly improves PFS and OS, with acceptable toxicity, for patients with uAGC.

Following the V325 trial, a multicenter phase III study (Chinese V325) utilizing reduced-dose DCF vs. CF for 243 patients with uAGC was conducted in China (9). Compared with CF, DCF significantly improved PFS (7.2 vs. 4.9 months, HR = 0.58), OS (10.2 vs. 8.5 months, HR = 0.71), and ORR (48.7% vs. 33.9%). In our study, comparing ivPOF with mFOLFOX6, PFS, OS, and ORR were 6.52 vs. 4.55 months (HR=0.56), 9.83 vs. 6.87 months (HR = 0.59), and 56.7% vs. 36.6%, respectively. Survival, which was numerically lower than in Chinese V325, may be explained by poorer PS among participants in our study, based on ECOG PS 0 or 1, respectively (ivPOF, 46.7% and 53.3%; mFOLFOX6, 33.3% and 66.7%). In the Chinese V325 study, PS was based on the Karnofsky Scale, such that scores ≥80 vs. <80, respectively, were as follows: DCF at 96.6% vs. 3.4% and CF at 93.9% vs. 6.1%. However, survival in the mFOLFOX6 group in our study was consistent with fluoropyrimidine plus oxaliplatin regimens in other Chinese investigator-initiated trials (5, 24). In terms of HR, PFS in our study is consistent with Chinese V325.

A randomized phase 3 trial with a triplet regimen of docetaxel, cisplatin, and S-1 did not improve survival compared to cisplatin and S-1 in previously untreated AGC. The reason may be that oral S-1 (which should be administered over 14 days every 21 days) replaced fluorouracil (which should be administered over 48 h). A longer medication-free interval benefits recovery in a strong triplet regimen, improving tolerance and preparing patients for the next treatment cycle. In this trial, dose intensity in the triplet group was relatively lower, which, consequently, may have impacted efficacy (25). In a randomized phase 2 study of docetaxel and oxaliplatin plus either capecitabine or fluorouracil, the capecitabine arm was worse for survival and response rate (26). Therefore, triplet regimens may benefit from continuous infusion fluorouracil with a shorter duration vs. oral fluoropyrimidine with a longer duration.

In the PHOENIX-GC study, IP paclitaxel failed to show a statistically significant improvement in survival; however, the IP paclitaxel dose is relatively low (20 mg/m^2^ days 1 and 8, every 3 weeks) compared with our study (80 mg/m^2^ day 1, every 2 weeks). The higher concentration gradient results in a higher rate of drug diffusion and anticancer effect (27). A recent prospective phase 2 study with oral capecitabine and IV oxaliplatin plus IP paclitaxel (40 mg/m^2^ days 1 and 8, every 3 weeks) showed improved survival compared with historical control (28). Therefore, we speculate that IP paclitaxel should improve survival, consistent with the PHOENIX-GC study, which showed a survival benefit in sensitivity analysis adjusted for baseline ascites (20), an effect that may be greater at higher doses.

Consistent with the V325 and Chinese V325 trials (8, 9), ORR was numerically higher for ivPOF vs. mFOLFOX6, such that more patients in the ivPOF group underwent curative treatment. A depth of only 1 µm in tumor nodules could be reached by IP paclitaxel (29). The diameter of target lesions to evaluate response was much larger than 1 µm according to RECIST criteria, precluding IP paclitaxel from taking effect. Therefore, it is understandable that the ORR for ipPOF is similar to that for mFOLFOX6. Additionally, in some patients, target lesions are outside the peritoneal cavity. Nonetheless, patients with relatively lower response rates may experience a survival benefit resulting from control of PM, which commonly appears as unmeasurable lesions, a main cause of death (3, 6, 7).

Conventional imaging technology to diagnose PM is the most common approach in daily clinical practice. However, staging laparoscopy detected more than 50% of cases with microscopic PM (including positive peritoneal cytology) for a locally advanced disease that was not detected by conventional imaging (30, 31). Systemic advanced disease may have a higher rate of microscopic PM. That may explain why patients who received IP triplet therapy had better PFS with or without MAPM (HR: 0.52 and 0.57, respectively) in forest plots. This phenomenon supports our observation that many patients without MAPM can benefit from IP paclitaxel. This finding emphasizes the need to develop more sensitive techniques to diagnose microscopic PM for IP treatment (32), because it is infeasible to perform staging laparoscopy for every patient.

From our clinical experience, we anticipated that both IV and IP paclitaxel would produce a good response in subjects with PM. PFS and OS with IV and IP paclitaxel in combination with mFOLFOX6 are numerically close, although our study is not powered to detect the difference. A study showed that paclitaxel concentration in ascites and plasma is similar 24 h after IV administration (33), which might support our hypothesis. Regarding the selection of ivPOF or ipPOF for uAGC, we suggest that ipPOF be considered when the predominant metastatic feature is a small mass in the peritoneum. Contrarily, for patients with a large mass or metastasis outside the abdominal cavity, ivPOF is preferable. Moreover, as an indwelling catheter may increase financial and provider burden, patient preference is an important consideration.

It is understandable that the frequency of AEs in the ipPOF arm is similar to that of mFOLFOX6, since IP administration reduces systemic toxicity (27). The frequency of AEs in ivPOF is also similar to that of mFOLFOX6, suggesting that higher response rates may relate to better performance status, hence patients’ ability to tolerate treatment. In a previous study, AEs were numerically lower in patients with docetaxel, oxaliplatin, and 5-FU (ORR: 46.6%) compared with docetaxel and oxaliplatin (23.1%) (26). Hematologic toxicity was the most common AE in the V325 and Chinese V325 studies, respectively: grade 3 or 4 neutropenia, 82% and 60.5%; febrile neutropenia, 29% and 13.4% in the DCF group. In our study, grade 3 or 4 neutropenia/febrile neutropenia rates were lower (30.0% and 3.3%, respectively, for ivPOF; 34.5% and 3.4%, respectively, for ipPOF), consistent with previous reports (4, 5, 10, 11, 15–20, 28). There was one subject in the ipPOF group who responded poorly to chemotherapy and experienced subcutaneous tumor implantation related to the indwelling catheter. It is unknown whether this would have occurred with better treatment response.

This study was terminated early due to slow accrual. The reasons for poor accrual were multiple and require further evaluation if this important patient-centered question is to be answered. Although the small sample size is a major limitation of this trial, given the prospective randomized design and planned patient treatment and follow-up, in addition to CIs on median OS and PFS and on the OS and PFS hazard ratios provided, this assumption should be reasonable. Further studies are warranted to confirm the superiority of ivPOF or ipPOF to mFOLFOX6 in a phase 3 setting with larger sample size. In our study, HER2-positive patients who might benefit from anti-HER2 treatment were not excluded. However, this cohort without anti-HER2 treatment did not reduce chemotherapeutic benefits. In addition, another limitation of this study is that the PD-L1 status is not available because this test was not a standard at the time of the trial.

Although contemporary treatment increasingly incorporates targeted or immunotherapy, the mainstay therapy for AGC continues to be chemotherapy combined with other modalities (23, 34, 35). Therefore, it is vitally important to evaluate and identify the most effective chemotherapeutic regimens. Adding IV or IP paclitaxel to mFOLFOX6 produced significantly improved PFS and OS with good tolerability as the first-line treatment of AGC. Although docetaxel-containing triplets are well-studied, paclitaxel and docetaxel are not identical in terms of structure, mechanism of action, or synergy with other agents; in particular, IV paclitaxel-based regimens are not more toxic compared with IV docetaxel (36). Moreover, a higher dose of IP paclitaxel appears to confer more clinical benefit, as shown in our study. Thus, further study of IV paclitaxel and high-dose IP paclitaxel-containing triplets is justified. To our knowledge, ours is the first randomized controlled multicenter study to evaluate an IV paclitaxel-containing triplet and is also the first to demonstrate the positive survival effect of an IP paclitaxel-containing regimen in previously untreated AGC.

## Data availability statement

The raw data supporting the conclusions of this article will be made available by the authors, without undue reservation.

## Ethics statement

The studies involving human participants were reviewed and approved by the central ethics committee of the Fujian Cancer Hospital. The patients/participants provided their written informed consent to participate in this study.

## Author contributions

RL and SZ had full access to all of the data in the study and take responsibility for the integrity of the data and the accuracy of the data analysis. All authors accessed and verified the data. All authors approved the final version of the manuscript. Study concept and design: RL, SZ, YC, NF, and XL. Acquisition, analysis, or interpretation of data: all authors. Drafting of the manuscript: RL, SZ, and LS. Critical revision of the manuscript for important intellectual content: all authors. Statistical analysis: RL, PL, WC, and WF. Obtained funding: RL and SZ. Administrative, technical, or material support: all authors. Study supervision: RL and SZ. All authors contributed to the article and approved the submitted version.

## Funding

This study was funded by the 2021 China Anti-Cancer Association-HengRui Anti-angiogenesis Targeted Tumor Research Fund (Grant No. 7).

## Acknowledgments

The authors thank the study participants, their families, investigators, site staff, and research teams who contributed to this study. The authors wish to acknowledge Caron Modeas, Evolved Editing, LLC, for editorial assistance.

## Conflict of interest

The authors declare that the research was conducted in the absence of any commercial or financial relationships that could be construed as a potential conflict of interest.

## Publisher’s note

All claims expressed in this article are solely those of the authors and do not necessarily represent those of their affiliated organizations, or those of the publisher, the editors and the reviewers. Any product that may be evaluated in this article, or claim that may be made by its manufacturer, is not guaranteed or endorsed by the publisher.
