# Supplementary material for: Phase 2 randomized controlled trial of intravenous or intraperitoneal paclitaxel plus mFOLFOX6 vs. mFOLFOX6 as first-line treatment of advanced gastric cancer
Source: Front Oncol. 2022 Sep 7;12:850242. doi: 10.3389/fonc.2022.850242 (PMC9491235; doi:10.3389/fonc.2022.850242)
Supplement: Supplementary Material S1 [file DataSheet_1.docx]

**Intravenous and/or intraperitoneal paclitaxel plus mFOLFOX6 versus mFOLFOX6 as first-line therapy for advanced gastric cancer: a randomized, open-label, phase 2 trial**

**Study design**

An open label, multicenter, randomized phase 2 clinical trial enrolling participants with histologically or cytologically confirmed metastatic or unresectable gastric or gastroesophageal junction adenocarcinoma with no previous systemic treatment. The study schema is displayed in Table 1.

**Table 1. Study Schema**


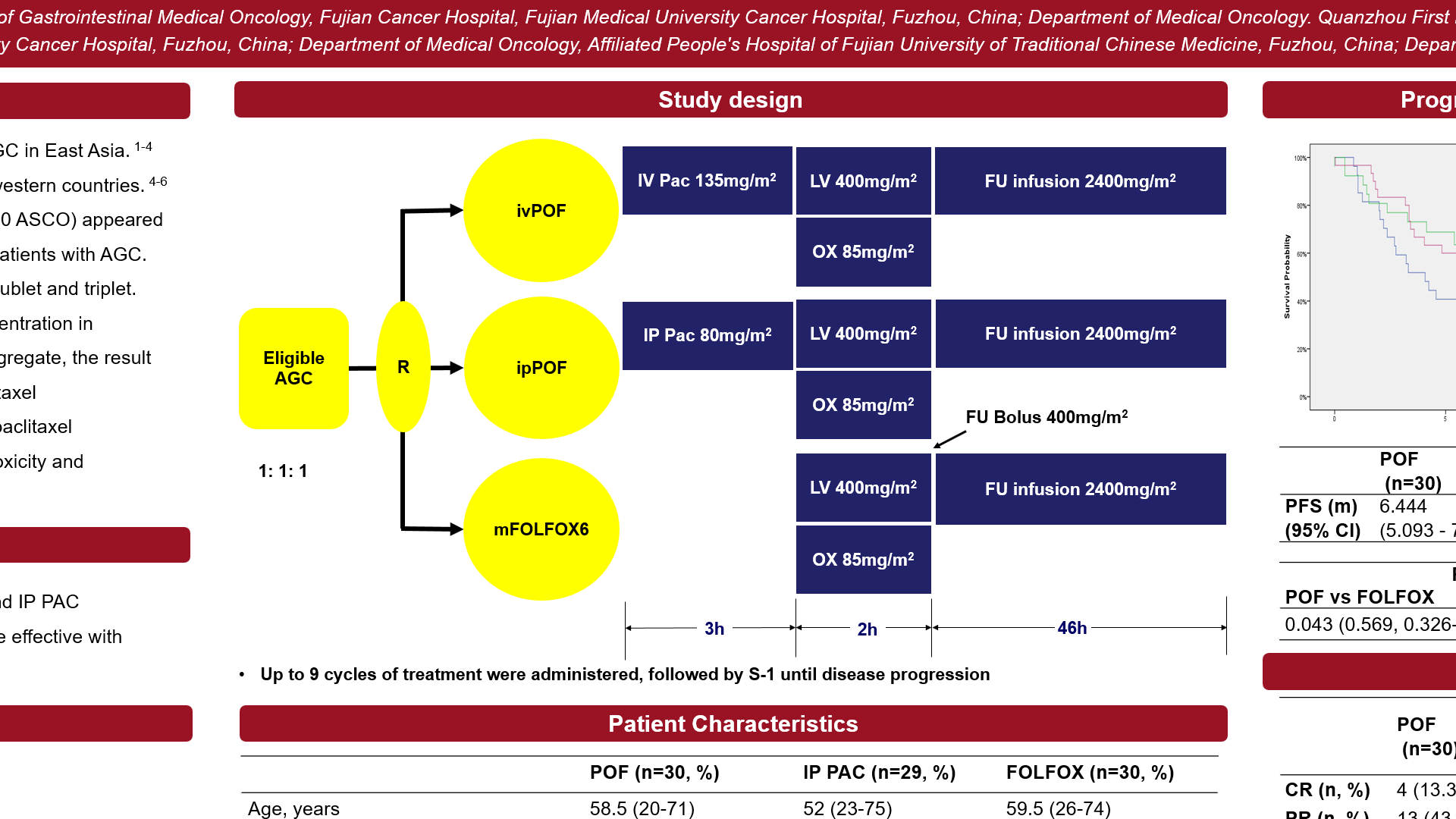


**Study setting**

This trial is planned to be conducted at approximately 10 oncology centers in China. Randomization, data collection, and analyses are performed centrally at Fujian Cancer Hospital.

**Study objectives**

**Primary objective**

The primary objective is to compare ivPOF (intravenous [IV] paclitaxel plus mFOLFOX6), ipPOF (intraperitoneal [IP] paclitaxel plus mFOLFOX6), and mFOLFOX6 for progression-free survival (PFS) in advanced gastric cancer (AGC).

**Secondary objectives**

Secondary objectives are to compare overall survival (OS), best overall tumor response per RECIST v1.1, adverse events (AEs) according to NCI-CTCAE v4.03, and quality of life parameters will be collected including EORTC QLQ-C30 and CIPN20 questionnaire among the three regimens.

**Participants and eligibility criteria**

Target accrual is 178 participants randomly assigned on a 1:1:1 basis to one of three study groups.

Inclusion criteria: histologically or cytologically confirmed metastatic or unresectable gastric or gastroesophageal junction adenocarcinoma with measurable disease according to Response Evaluation Criteria in Solid Tumors (RECIST, version 1.1; written informed consent prior to any protocol-related procedure (including screening evaluation); no prior chemo- or radiation therapy except neo- or adjuvant chemotherapy completed ≥6 months prior to relapse; age 18 to 75 years; Eastern Cooperative Oncology Group performance status (ECOG PS) 0 or 1; life expectancy ≥3 months; adequate organ function and lab values (platelets ≥100 x 10^9^/L, hemoglobin ≥9.0 g/dl, absolute neutrophils ≥1.5 x 10^9^/L, serum bilirubin ≤1.5 x ULN; AST ≤2.5 x ULN [except ≤5 x ULN if liver metastases are present]), and willingness and ability to comply with study visits, treatment plans, laboratory tests, response assessment, and all other study-related procedures. Women of childbearing potential must have a negative serum or urine pregnancy test within one day of study entry and agree to use acceptable contraceptive methods during study participation and for 6 months thereafter.

Exclusion criteria: pregnant or lactating women; ascites requiring frequent drainage; peripheral neuropathy grade ≥2 according to National Cancer Institute Common Terminology Criteria for Adverse Events (NCI-CTCAE) version 4.03; brain or leptomeningeal involvement; concurrent cancer; uncontrolled significant comorbidities; history of hypersensitivity to any of the study agents; participation in another clinical study with an investigational product during the last 30 days before inclusion; or patients who are deemed unsuitable for the study by the investigators.

As trastuzumab is not covered by medical insurance and is unaffordable for most Chinese patients, those who do not intend to use it may enter this study regardless of human epidermal growth factor receptor 2 (HER2) status. Peritoneal metastasis, diagnosed by previous laparoscopy or laparotomy, ultrasound, computed tomography, magnetic resonance imaging, positron emission tomography, or ascites, is not an eligibility criterion.

**Randomization and blinding**

Before the start of the study, a computer-generated sequence of random numbers were placed in a series of plain, sealed envelopes with patient numbers on them by the research nurse. These envelopes were created and kept at the School of Public Health of Fujian Medical University, and only opened at the time of subject randomization, again by the research nurse. Individuals directly involved in the study had no access to these envelopes. Eligible participants will be randomly assigned (1:1:1 ratio) to one of the three intervention groups (ivPOF, ipPOF, or mFOLFOX6). There are no stratification factors.

The statistician and research nurse are blinded to the recruitment procedure prior to study initiation. Because this is an open-label trial, participants and physicians are not blinded to the study groups. A site radiologist who assesses tumor response, and a statistician who analyzes the data are blinded to the study groups.

**Interventions**

ivPOF regimen:

A 3-hour intravenous infusion of paclitaxel 135 mg/m^2^ followed by mFOLFOX6, omitting fluorouracil bolus (oxaliplatin 85 mg/m^2^ plus leucovorin 400 mg/m^2^, administered simultaneously over a 2-hour infusion period, followed by a 46-hour infusion of 5-florouracil 2400 mg/m^2^).

ipPOF regimen:

A 3-hour intraperitoneal infusion of paclitaxel 80 mg/m^2^ followed by mFOLFOX6, omitting fluorouracil bolus (oxaliplatin 85 mg/m^2^ plus leucovorin 400 mg/m^2^, administered simultaneously over a 2-hour infusion period, followed by a 46-hour infusion of 5-florouracil 2400 mg/m^2^).

A central venous catheter is to be indwelled into the abdominal cavity before administration of IP paclitaxel, which is diluted in normal saline 500 mL and administered over 3 hours. Normal saline perfusion 500 mL is to be given before and after paclitaxel (total 1000 mL) but should be reduced for ascites, accordingly. The catheter is removed two days after treatment administration.

mFOLFOX6 regimen:

A 2-hour infusion of oxaliplatin 85 mg/m^2^ plus leucovorin 400 mg/m^2^ followed by a fluorouracil bolus 400 mg/m^2^ and a 46-hour infusion of fluorouracil 2400 mg/m^2^.

For all regimens, treatment is repeated every 14 days for up to 9 cycles, with maintenance therapy consisting of S-1 80 mg/m^2^ days 1-14 every 3 weeks. Treatment is continued until progressive disease (PD), unacceptable toxicity, subject refusal, or investigator decision. Antiemetic prophylaxis is to be given according to local protocols; granulocyte colony-stimulating factor is not recommended as primary prophylaxis. Pre-medications (antihistamine, corticosteroid, and H2 receptor antagonist) are to be administered for prophylaxis of hypersensitivity reactions to IV or IP paclitaxel.

**Treatment modification**

To ensure standardization across study sites and treatment regimens, dose delay should be carried out before dose modification as detailed in Tables 2 and 3. Any treatment delay or dose modification must be truthfully and completely detailed in the medical record and CRF.

**Dose delay**

For a grade 3 or 4 hematological or non-hematological AE, treatment delay should be implemented according to Table 2. The duration of treatment delay in any cycle shall not exceed 14 days to insure adequate drug dose intensity. If a delay exceeds 14 days, the participant is discontinued from protocol therapy, although tumor evaluation will still be carried out.

**Dose modification**

Guidelines for dose modification are provided in Table 3. In principle, dose adjustments should be carried out according to the AE spectrum of the drug, and specific modifications or delays should be according to the judgment of the investigator and best clinical practice. If a dose is reduced, it is not to be returned to the previous level.

**Table 2. Treatment delay**

* Grading according to NCI-CTC AE 4.03

| Toxicity | Paclitaxel | Oxaliplatin | Fluorouracil |
| --- | --- | --- | --- |
| Grade 3 Hematological toxicity (non-leukopenia) | Hold until NCI CTCAE ≤grade 1; resume at same dose. If NCI CTCAE ≥grade 3 again, resume at reduced dose. | Hold until NCI CTCAE ≤grade 1; resume at same dose. If NCI CTCAE ≥grade 3 again, resume at reduced dose. | Hold until NCI CTCAE ≤grade 1, resume at same dose. If NCI CTCAE ≥grade 3 again, resume at reduced dose. |
| Grade 4 Hematological toxicity (non-leukopenia) | Hold until NCI CTCAE ≤grade 1; resume at reduced dose. | Hold until NCI CTCAE ≤grade 1; resume at reduced dose. | Hold until NCI CTCAE ≤grade 1, resume at reduced dose. |
| Grade 3 or 4 Leukopenia | Hold until NCI CTCAE ≤grade 2; dose reduction at investigator’s discretion. | Hold until NCI CTCAE ≤grade 2; dose reduction at investigator’s discretion. | Hold until NCI CTCAE ≤grade 2; dose reduction at investigator’s discretion. |
| Grade 3 Neurotoxicity | Permanently discontinue | Permanently discontinue | Permanently discontinue |
| Grade 3 Diarrhea, Stomatitis,  Hand/foot syndrome | Hold until NCI CTCAE ≤grade 1; resume at same dose. | Hold until NCI CTCAE ≤grade 1; resume at same dose. | Hold until NCI CTCAE ≤grade 1; resume at same dose. If NCI CTCAE ≥grade 3 again, resume at reduced dose. |
| Grade 4 Diarrhea, Stomatitis,  Hand/foot syndrome | Hold until NCI CTCAE ≤grade 1; resume at same dose. | Hold until NCI CTCAE ≤grade 1; resume at same dose. | Hold until NCI CTCAE ≤grade 1; resume at reduced dose. |
| Grade 3  Allergy | Investigator decision whether to continue. | Investigator decision whether to continue. | Investigator decision whether to continue. |
| Grade 4  Allergy | Permanently discontinue | Permanently discontinue | Permanently discontinue |
| Other drug- related  Grade 3 or 4 toxicities | Hold until NCI CTCAE ≤grade 1 (for elevated transaminase with liver metastases, hold until NCI CTCAE ≤grade 2); resume at same dose. If NCI CTCAE ≥grade 3 again, resume at reduced dose. | Hold until NCI CTCAE ≤grade 1 (for elevated transaminase with liver metastases, hold until NCI CTCAE ≤grade 2); resume at same dose. If NCI CTCAE ≥grade 3 again, resume at reduced dose. | Hold until NCI CTCAE ≤grade 1 (for elevated transaminase with liver metastases, hold until NCI CTCAE ≤grade 2); resume at same dose. If NCI CTCAE ≥grade 3 again, resume at reduced dose. |

**Table 3. Dose reduction**

| Drug | Initial dose level | First dose reduction | Second dose reduction |
| --- | --- | --- | --- |
| IV Paclitaxel  IP Paclitaxel  Oxaliplatin  Fluorouracil | 135 mg/m^2^  80 mg/m^2^  85 mg/m^2^  2400 mg/m^2^ | 120 mg/m^2^  70 mg/m^2^  75 mg/m^2^  2000 mg/m^2^ | No further reduction; delay treatment until toxicity resolves |

**Study visits and data collection**

Table 4 details study visits and assessments.

Baseline data to be collected within 7 days prior to treatment initiation:

1. signed informed consent form;
2. medical history;
3. physical examination and vital signs including body temperature, breathing, heart rate, blood pressure, and weight;
4. electrocardiogram;
5. ECOG-PS;
6. complete blood counts;
7. urinalysis;
8. liver, kidney function and electrolytes tests;
9. Carcinoma Embryonic Antigen;
10. pregnancy test (if applicable);
11. CT, MRI or PET-CT imaging;
12. ECTOG QLQ-C30 and CIPN 20;
13. adverse events.

During study participation, data to be collected 1-2 days prior to every cycle after the first cycle:

- 1. physical examination and vital signs including body temperature, breathing, heart rate, blood pressure, and weight;
  2. ECOG-PS;
  3. complete blood counts;
  4. urinalysis;
  5. liver, kidney function and electrolytes tests;
  6. adverse events;
  7. dose delays or modifications;
  8. concomitant treatment.

During study participation, data to be collected every 6 weeks (within 1-2 days):

- 1. electrocardiogram;
  2. Carcinoma Embryonic Antigen;
  3. CT, MRI or PET-CT imaging;
  4. EORTC QLQ-C30 and CIPN-20.

**Table 4. Study visits and assessments.**

| **Procedure/**  **Assessment** | **Screening 7 days before C1D1**  **Initiation** | **Treatment**  **every 14 days ± 2 days** | | | | **End of treatment** | **Maintenance every 6 weeks ± 7 days** | **Follow-up**  **post PD every 3 months ±7 days*** |
| --- | --- | --- | --- | --- | --- | --- | --- | --- |
|  |  | **C1** | | **C2-9** | |  |  |  |
|  |  | D1 | D8 | D1 | D8 |  |  |  |
| Informed consent | **ⅹ** |  |  |  |  |  |  |  |
| Demographics | **ⅹ** |  |  |  |  |  |  |  |
| Medical history | **ⅹ** |  |  |  |  |  |  |  |
| Vital signs, physical examination | **ⅹ** |  |  | **ⅹ** |  | **ⅹ** | **ⅹ** |  |
| ECG | **ⅹ** | **Every 6 weeks** | | | | **ⅹ** |  |  |
| ECOG PS | **ⅹ** |  |  | **ⅹ** |  | **ⅹ** | **ⅹ** |  |
| Complete blood counts | **ⅹ** | **ⅹ** |  | **ⅹ** |  | **ⅹ** |  |  |
| Urinalysis | **ⅹ** | **ⅹ** |  | **ⅹ** |  | **ⅹ** |  |  |
| Liver & renal function and electrolyte test | **ⅹ** | **ⅹ** |  | **ⅹ** |  | **ⅹ** |  |  |
| CEA | **ⅹ** | **Every 6 weeks** | | | | **ⅹ** | **ⅹ** |  |
| CT/MRI/PEC-CT | **ⅹ** | **Every 6 weeks** | | | | **ⅹ** | **ⅹ** |  |
| Pregnancy test (if applicable) | **ⅹ** |  |  |  |  |  |  |  |
| QLQ-C30&CIPN20 | **ⅹ** | **Every 6 weeks** | | | | **ⅹ** | **ⅹ** |  |
| Adverse events | **ⅹ** | **ⅹ** |  | **ⅹ** |  | **ⅹ** | **ⅹ** |  |
| Dose delay and/or modification |  |  |  | **ⅹ** |  |  |  |  |
| Concomitant treatment |  |  |  | **ⅹ** |  | **ⅹ** |  |  |

* For assessment of survival and information of subsequent treatment

**Data collection**

Electronic case report forms (eCRFs) will be used to record data for all study participants including those who fail to complete the trial. Reason for withdrawal must be recorded in the eCRF. Except for withdrawal of consent, all participants who are discontinued from the trial will be followed for safety until the study is completed.

**Outcome measures**

*Primary outcome*

Progression-free survival is defined as time from treatment assignment to documented PD per RECIST version 1.1 or death resulting from any cause, whichever occurs first.

*Secondary outcomes*

1. Overall survival is defined as time from treatment assignment to death from any cause.
2. Best overall tumor response is from baseline per RECIST version 1.1.
3. Adverse events are graded according to the NCI-CTCAE version 4.03.
4. Quality of life is assessed by EORTC QLQ-C30 and QLQ-CIPN 20.

**Treatment emergent adverse events**

Treatment emergent adverse events (TEAEs) are graded according to CTCAE version 4.03 guidelines. A TEAE is characterized in terms of severity, causality, toxicity grading, and possible etiologies in order to determine whether they meet the criteria for a serious adverse event (SAE). All ongoing TEAEs must be recorded and followed until resolved to baseline or deemed irreversible, unless the participant is lost to follow-up or withdraws consent.

Any SAE or AE of special interest, any case of drug overdose, or any case of pregnancy while on study will be reported to the Chinese Ethics Committee of Registering Clinical Trials and communicated to local Ethics Committees and study sites within 24 hours of awareness. Standard operating procedures will be activated to adjust or potentially terminate the trial in accordance with ICH Good Clinical Practice.

**Criteria for participant discontinuation**

(1) Intolerable AEs.

(2) Subject (or legal representative) voluntarily decides to discontinue participation for any reason.

(3) Investigator determines that the risk/benefit ratio is unacceptable.

(4) Provider determination based on clinical considerations.

(5) The participant becomes pregnant.

**Statistical analysis plan**

The sample size is based on previous studies in which median PFS was 7 months for ivPOF and ipPOF and was 4 months for mFOLFOX. It is calculated by a log-rank test based on the primary outcome, to verify superiority of ivPOF or ipPOF over mFOLFOX6 in AGC. Using a one-sided α of 0.05 with 0.025 allocated to the hypothesis of superiority of either ivPOF or IPPOF over mFOLFOX, we calculate that 54 participants/group are needed over 24 months of accrual and 24 months of follow-up, to achieve 80% statistical power for each hypothesis. Considering a dropout rate of 10%, the total number to be enrolled is 178. The dataset for efficacy and safety analyses will include all randomly assigned patients who receive at least one dose of study medication. Categorical variables are presented as frequencies/proportions and continuous variables as medians/interquartile ranges (IQRs). Between-group differences are analyzed with the χ2 test, Fishers’ exact test, and Mann-Whitney U test. P<0.05 is considered statistically significant. We estimate PFS and OS using Kaplan-Meier with a P-value. Hazard ratios and 95% confidence intervals (CIs) are estimated using Cox proportional hazards model. Data are analyzed using R software v. 4.0.
